# A New Uterine Dilator

**Published:** 1879-04

**Authors:** Eli Griffin

**Affiliations:** Atlanta, Ga.


### A NEW UTERINE DILATOR.

By ELI GRIFFIN, M.D., Atlanta, Ga.

It is with hesitation that I report to the profession, through your Journal, this simple instrument on account of the wide-spread prevalence of the morbid diposition of these days to neglect the acquirement of skill and perfection in the manipulation of existing instruments, to discover some modification that may palliate some individual lack of skill or operative ingenuity. Yet I would not be understood to condemn necessary improvements, but every one is acquainted with this tendency of the times—a mania to herald their name in connection with some invention. In consideration, however, of the simplicity, convenience and cheapness of this dilator, I have thought that some other might derive the benefit from its use that I have in times past.

The instrument consists simply of a of plate of steel, with a breadth adapted to the anatomy of the cervical canal. The edges of this are turned in on themselves, so as to present a round surface to the uterine wall instead of a sharp edge. The steel is bent into two equal arms, presenting in its relaxed state, a V shaped appearance. The points of these two arms are rounded off in the same manner as the edges, which renders it no more irritating than any other stem pressary, with the exception of what may arise from its dilating capacity. The introduction is accomplished by seizing the spring in a pair of strong forceps, or ordinary dressing forceps, and passing it well up into the canal, or to the depth required by the individual case. It may be introduced by either end indifferently; I have used it both ways with equal benefit and success.

The advantages that it presents over the sponge tent and allied agents, are its convenience and durability, with the greater ease and safety with which it may be employed. In comparison with other dilators, it presents the advantages of being self-sustaining and handy, as it can be introduced into the uterus and left there as long as necessary without the intervention of manual assistance. Its dilating capacity may be increased by using thicker plates, until it be of strength sufficient for any occasion. Increased dilation of the external or internal as may be accomplished individually, by introducing one or the other end of the instrument foremost.

In conclusion, I would beg each one who may have occasion to institute gradual dilatation of the cervical canal to try this instrument. I have certainly derived great benefit from it in my own practice, and as for its merits, therefore, in my own belief, I unhesitatingly recommend it for the cases to which it is adapted.

